# S10 How can HEPA research contribute to shifting modes of transport towards active mobility?

**DOI:** 10.1093/eurpub/ckae114.240

**Published:** 2024-09-26

**Authors:** Antonia Tcymbal, Karim Abi-Omar

**Affiliations:** Friedrich-Alexander Universität Erlangen-Nürnberg; Department of Sport Science and Sport, Friedrich-Alexander-Universität Erlangen-Nürnberg, Germany

## Abstract

Active mobility represents a win-win for individual and planetary health. With this in mind, the question on how to shift modes of transport towards walking and biking becomes an increasingly urgent one for HEPA research. The symposium presents different approaches on how to foster active transport: the use of GIS data to identify bikeable neighborhoods for adolescent; the HEAT – a widely used tool that enables to calculate the economic value of active mobility; a science-based project on the determinants of biking among children; and an interdisciplinary collaboration to explore how the transition towards active mobility can be accelerated.

By integrating science- and practice-based presentations, the symposium will provide attendees with an excellent opportunity to reflect on the complexity of fostering active mobility. It will also enhance participants’ understanding on how active mobility is linked to planetary health and how research-driven approaches can address transport choices.

